# Longitudinal study of the early-life fecal and nasal microbiotas of the domestic pig

**DOI:** 10.1186/s12866-015-0512-7

**Published:** 2015-09-21

**Authors:** Mackenzie Jonathan Slifierz, Robert M. Friendship, J. Scott Weese

**Affiliations:** Department of Pathobiology, Ontario Veterinary College, University of Guelph, Guelph, ON Canada; Department of Population Medicine, Ontario Veterinary College, University of Guelph, Guelph, ON Canada

## Abstract

**Background:**

The mammalian microbiota plays a key role in host health and disease susceptibility. However, knowledge of the early-age microbiota of pigs is lacking. The purpose of this study was to use high-throughput next-generation sequencing to characterize the fecal and nasal microbiotas of pigs during early life.

**Results:**

Ten commercially-raised pigs were randomly enrolled at birth and sampled throughout the first 7 weeks of life. DNA was extracted from fecal and nasal samples and the hypervariable region V4 of the 16S rRNA gene was amplified. The product was sequenced using the Illumina MiSeq platform and 2 × 250 chemistry. Sequencing data was processed and analyzed with the mothur algorithms using an operational taxonomic unit approach. In total, 4.7 million and 5.4 million high-quality sequences were recovered from fecal and nasal samples, respectively. Analysis revealed that these microbiotas contain a very rich and diverse population of bacteria that display a remarkable evolution during the first 7 weeks of life. During this developmental period, a pig was exposed to an average of 1,976 and 6,257 species of bacteria by way of the gastrointestinal and respiratory tracts, respectively. Aging was significantly associated with an increasing measure of richness and diversity as well as with distinct changes to the core microbiota. At 2–3 weeks post-weaning, the rapidly developing microbiotas appeared to reach a developmental milestone as a relative degree of stability was evident.

**Conclusions:**

Pigs are exposed to an incredibly rich and diverse mixture of bacteria during early-life as demonstrated by next-generation sequencing methodology. These findings expand the knowledge of the developing porcine microbiota which is important for understanding susceptibility to disease, particularly for vulnerable neonatal pigs.

**Electronic supplementary material:**

The online version of this article (doi:10.1186/s12866-015-0512-7) contains supplementary material, which is available to authorized users.

## Background

The microorganisms that populate the body, collectively known as the microbiota, are important drivers of host health and metabolism. Characterization of the porcine microbiota using previous (primarily culture-dependent) techniques provides only a narrow understanding of the complexity of these ecosystems due to methodological limitations [1, 2]. Advancements in next-generation sequencing and bioinformatics have only recently given researchers the opportunity to examine the composition and diversity of these microbial populations in significant detail. Presently, high-throughput sequencing of the hypervariable regions of the 16S rRNA gene can provide a depth of coverage that is far greater than any previous method [3].

The microbiotas of the gastrointestinal and respiratory tracts of pigs are of particular interest due to the association of these body sites with common swine diseases or pathogens. These microbiotas provide a first line of defense against foreign invaders as competition and interaction between bacteria can protect a host from becoming colonized with particular pathogens [4]. However, the developing microbiota of young pigs is particularly vulnerable to disruptions which can result in long-term impacts that may affect disease susceptibility and growth performance [5]. Such variation in the porcine microbiota has been associated with stress, diet, management practices, and antimicrobial compounds [5–7].

Previous research exploring the gut microbiota in adult pigs using next-generation sequencing methods has revealed an incredibly diverse population of bacteria [8]. The adult porcine gut microbiota has been found to contain at least 7 identifiable bacterial phyla, the predominant being Firmicutes and Bacteroidetes, and at least 171 genera of bacteria [8]. Similarly, the nasal cavity of adult pigs also harbours an incredibly diverse and rich microbial ecosystem which contains an estimated 1,749 species of bacteria from 124 different genera. The porcine nasal microbiota was found to contain 9 phyla of bacteria, of which Proteobacteria, Firmicutes and Spirochaetes predominated [7].

However, despite the emerging understanding of the microbiota of adult pigs, characterization of the development of the early-age microbiota using next-generation techniques is lacking. Further understanding of the fecal and nasal microbiotas can provide significant insight into swine health and susceptibility to disease, especially among young pigs who are particularly vulnerable. Therefore, the objective of this longitudinal investigation was to characterize the transformation of the fecal and nasal microbiotas of conventionally-raised pigs throughout the first 7 weeks of life using high-throughput next-generation sequencing.

## Results

### Fecal microbiota

**Fig. 1:**
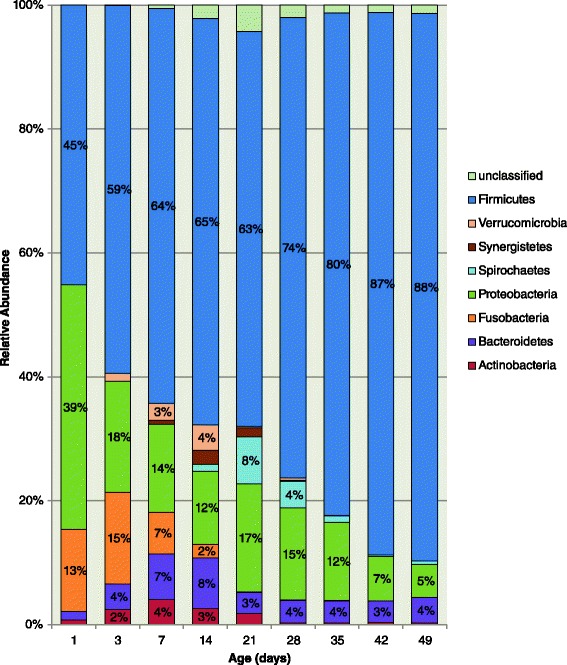
Age-associated change in relative abundance of bacterial phyla from the feces of young pigs (n = 10)

There were a total of 4,711,191 sequences recovered from 90 fecal samples after removal of erroneous and poor quality reads. The median number of sequences per sample was 47,628 (Range: 9,725–100,859). Sequences recovered from the feces of 10 pigs over the 7-week period clustered into 6,714 OTUs. The median number of OTUs recovered per pig during the study period was 1976 (Range: 1,600–2,112). The OTUs were classified into 19 bacterial phyla and 489 genera, although only five phyla had >1 % overall relative abundance: Firmicutes (70 %), Proteobacteria (16 %), Bacteroidetes (4.3 %), Fusobacteria (1.6 %), and Actinobacteria (1.4 %). Only 1.4 % of sequences were unclassified at the phylum level. However, the relative abundance of bacterial phyla varied considerably with age (Fig. 1). Specifically, aging was associated with a greater relative abundance of Firmicutes (*P* < 0.001), and lower relative abundance of Proteobacteria (*P* < 0.001), Fusobacteria (*P* < 0.001), and Actinobacteria (*P* < 0.001). The most dominant genera during the pre-weaning phase were *Clostridium* and *Escherichia*, while *Megasphaera* and *Lactobacillus* dominated the post-weaning phase (Table 1).

**Table 1:** Relative abundance of the top five predominant classes and genera during each period of development

Age Range (days)	Dominant classes (Mean relative abundance, %)		Dominant genera (Mean relative abundance, %)	
	Fecal	Nasal	Fecal	Nasal
1–3	Clostridia (44.5)	Gammaproteobacteria (37.7)	*Clostridium* (17.9)	*Moraxella* (24.2)
	Gammaproteobacteria (27.1)	Bacilli (27.5)	*Escherichia* (15.0)	*Rothia* (14.9)
	Fusobacteria (14.0)	Actinobacteria (17.6)	*Fusobacterium* (10.5)	*Clostridium* (10.4)
	Bacilli (6.0)	Clostridia (13.7)	*Clostridium* XIVa (4.3)	*Globicatella* (9.9)
	Bacteroidia (2.8)	Betaproteobacteria (1.5)	*Lactobacillus* (4.2)	*Actinobacillus* (5.3)
7–21	Clostridia (44.5)	Gammaproteobacteria (49.3)	*Clostridium* (8.8)	*Moraxella* (46.7)
	Gammaproteobacteria (10.8)	Clostridia (27.9)	*Escherichia* (8.6)	*Clostridium* (16.5)
	Bacilli (10.4)	Bacilli (11.1)	*Lactobacillus* (8.2)	*Lactobacillus* (3.9)
	Bacteroidia (5.9)	Betaproteobacteria (3.2)	*Clostridium* XIVa (7.4)	unclassified Firmicutes (3.4)
	Erysipelotrichia (4.1)	Actinobacteria (2.8)	unclassified Firmicutes (5.2)	*Kingella* (2.8)
28–35	Clostridia (29.1)	Gammaproteobacteria (47.5)	*Megasphaera* (14.0)	*Moraxella* (40.0)
	Negativicutes (26.5)	Bacilli (27.9)	*Lactobacillus* (12.3)	*Lactobacillus* (21.2)
	Bacilli (15.6)	Clostridia (17.1)	*Clostridium* (4.6)	*Blautia* (3.0)
	Gammaproteobacteria (6.7)	Erysipelotrichia (2.9)	unclassified Firmicutes (4.2)	Erysipelotrichaceae incertae sedis (2.9)
	Erysipelotrichia (4.2)	Betaproteobacteria (1.0)	*Succinivibrio* (4.1)	*Staphylococcus* (2.4)
42–49	Negativicutes (32.4)	Gammaproteobacteria (59.5)	*Megasphaera* (21.2)	*Moraxella* (56.5)
	Clostridia (31.7)	Bacilli (15.7)	*Lactobacillus* (12.3)	*Lactobacillus* (11.1)
	Bacilli (17.4)	Clostridia (15.1)	*Roseburia* (4.2)	*Kingella* (3.2)
	Erysipelotrichia (5.4)	Betaproteobacteria (3.4)	unclassified Firmicutes (3.9)	unclassified Firmicutes (2.9)
	Bacteroidia (3.7)	Erysipelotrichia (2.2)	Erysipelotrichaceae incertae sedis (3.9)	*Blautia* (2.3)

**Fig. 2:**
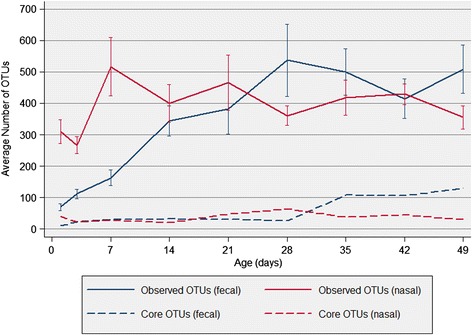
Observed and core OTUs in subsampled fecal and nasal samples from young pigs. In this analysis, core OTUs were defined as observed OTUs shared between all pigs at a given time point (no relative abundance threshold)

**Fig. 3:**
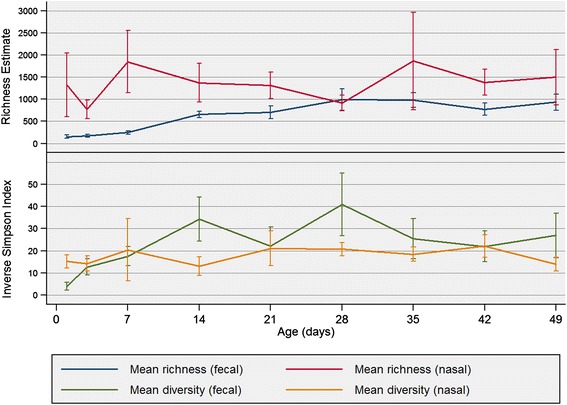
Estimated richness (CatchAll) and diversity (Inverse Simpson index) of fecal and nasal samples from pigs

Random subsampling of 9,725 sequences was completed for each fecal sample to normalize sequence numbers. The median sample richness was 665 OTUs (Range: 73–1,562), although there was significant age-related variation as richness was positively correlated with age (ρ = 0.79, *P* < 0.001). The median inverse Simpson’s diversity index was 19.9 (Range: 1.1–72.1) and diversity positively correlated with age (ρ = 0.48, *P* < 0.001). The age-related change in the number of observed and core OTUs is presented in Fig. 2, while measures of richness and diversity are shown in Fig. 3.

**Fig. 4:**
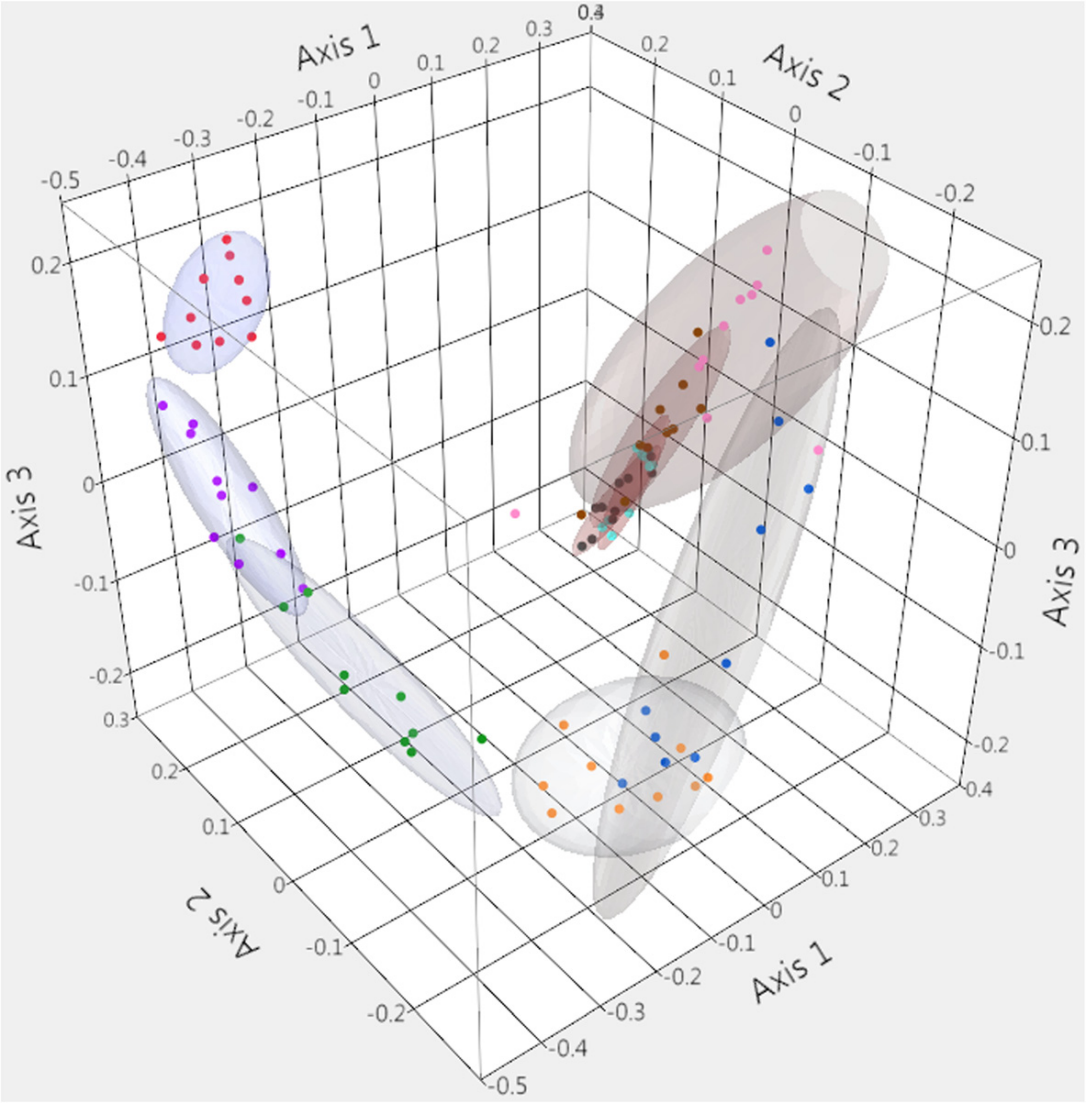
Three-dimensional principal coordinates analysis of the community membership of the porcine fecal microbiota (Jaccard Index). Coloured points and ellipses indicate age groups: 1 (red), 3 (violet), 7 (green), 14 (orange), 21 (blue), 28 (pink), 35 (brown), 42 (grey), 49 (indigo) days of age

The community membership (Jaccard measure of dissimilarity) of the fecal microbiota is visually represented in Fig. 4. There was distinctive clustering of samples according to age group and AMOVA analysis of the Jaccard indices demonstrated significantly differences between all age groups (*P* < 0.05). However, while statistically different, by 2-weeks post-weaning there was identifiable overlap between age groups suggesting development of a relatively more stable community membership. Additionally, community structure (Yue and Clayton measure of dissimilarity) is visualized as a dendrogram in Additional file 1: Figure S1. Yue and Clayton indices were significantly different between all age groups (*P* < 0.05) with the exception of 35-day-old and 42-day-old pigs (*P* = 0.293). There was also strong clustering of samples according to phase of production (pre-weaning and post-weaning) for both community membership (*P* < 0.001) and community structure (*P* < 0.001).

**Fig. 5:**
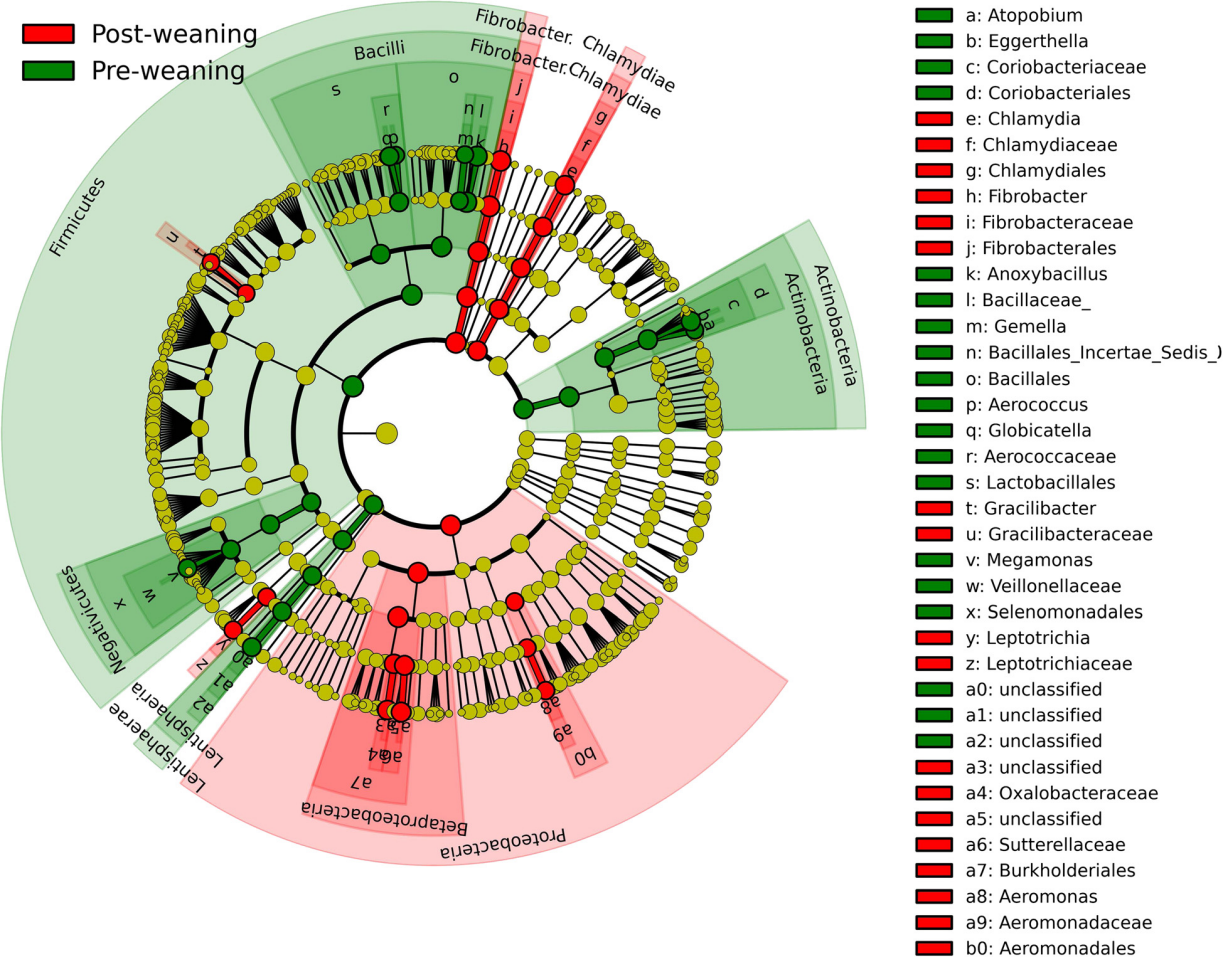
Cladogram of bacterial fecal biomarkers associated with phase of production (LEfSe). Samples were grouped into pre-weaning (days 1–21) or post-weaning (days 28–49). This hierarchal tree of taxonomical nodes, where diameter of the nodes indicates relative abundance, shows fecal biomarkers for the pre-weaning and post-weaning phases

The core fecal microbiota was notably dichotomized between the pre-weaning and post-weaning phases of production (Table 2). Probabilistic modelling using DDM demonstrated the presence of two metacommunities (enterotypes), with 96 % (48/50) of fecal samples from suckling pigs being partitioned into one community type and 95 % (38/40) of fecal samples from weaned pigs being partitioned into another community type (*P* < 0.001). Analysis using the LEfSe algorithm (LDA log score threshold = 2) also revealed 26 OTUs characteristic of the pre-weaning stage and 24 OTUs characteristic of the post-weaning stage. The pre-weaning indicator OTUs clustered into the bacterial classes Bacilli, Negativicutes, Actinobacteria and Lentisphaeria, while the post-weaning indicator OTUs clustered into the classes Betaproteobacteria, Fibrobacteria, and Chlamydiae (Fig. 5).

**Table 2:** The genus-level taxonomy of abundant core OTUs of the porcine fecal and nasal microbiotas

Age (days)	Genera of the core OTUs* of the porcine microbiotas (% of pigs with OTU)	
	Fecal Microbiota	Nasal Microbiota
1	*Escherichia* (Shigella) (100), *Clostridium sensu stricto* (100)	*Globicatella* (100), *Haemophilus* (100), *Clostridium sensu stricto* (100), *Rothia* (80), *Staphylococcus* (80)
3	*Clostridium sensu stricto* (100), *Clostridium* XIVa (80), *Haemophilus* (80), *Lachnospiracea incertae sedis* (80)	*Globicatella* (100), *Rothia* (100), *Haemophilus* (80), *Clostridium sensu stricto* (80)
7	*Clostridium sensu stricto* (80), *Clostridium* XIVa (80), *Desulfovibrio* (80)	*Clostridium sensu stricto* (80)
14	*Escherichia* (Shigella) (100), *Clostridium* XIVa (80)	*Clostridium sensu stricto* (80), *Kingella* (80), unclassified Firmicutes (80)
21	None	*Clostridium sensu stricto* (100), *Kingella* (80), unclassified Firmicutes (80)
28	*Megasphaera* (80), *Lactobacillus* (80), *Acidaminococcus* (80)	*Lactobacillus* (100), *Erysipelotrichaceae incertae sedis* (100), *Enterococcus* (100), *Haemophilus* (80), *Blautia* (80)
35	*Megasphaera* (100), *Lactobacillus* (100), *Butyricicoccus* (100), *Erysipelotrichaceae incertae sedis* (80), *Selenomonas* (80), *Roseburia* (80), *Acidaminococcus* (80), *Faecalibacterium* (80), unclassified Firmicutes (80)	*Lactobacillus* (100)
42	*Megasphaera* (100), *Butyricicoccus* (100), *Erysipelotrichaceae incertae sedis* (100), *Roseburia* (100), *Acidaminococcus* (100), *Faecalibacterium* (100), *Lactobacillus* (80), *Selenomonas* (80), *Streptococcus* (80), *Prevotella* (80), *Clostridium* XI, unclassified Firmicutes (80)	*Lactobacillus* (100), *Sarcina* (100), *Erysipelotrichaceae incertae sedis* (80), *Kingella* (80), unclassified Firmicutes (80)
49	*Megasphaera* (100), *Butyricicoccus* (100), *Erysipelotrichaceae incertae sedis* (100), *Roseburia* (100), *Lactobacillus* (80), *Streptococcus* (80), *Prevotella* (80), *Gemmiger*(80)	*Lactobacillus* (80), unclassified Firmicutes (80)

* Present at >1 % relative abundance

The most common core OTUs (>1 % relative abundance in ≥80 % of pigs) prior to weaning were from Clostridia (*Clostridium sensu stricto* and *Clostridium* cluster XIVa). Interestingly, no core OTUs with >1 % relative abundance could be identified at 21 days of age. However, after weaning, 3 core OTUs were present throughout the entire nursery phase at >1 % relative abundance in at least 80 % of pigs: *Megasphaera*, *Lactobacillus*, and Erysipelotrichaceae *incertae sedis* (Table 2).

### Nasal microbiota

**Fig. 6:**
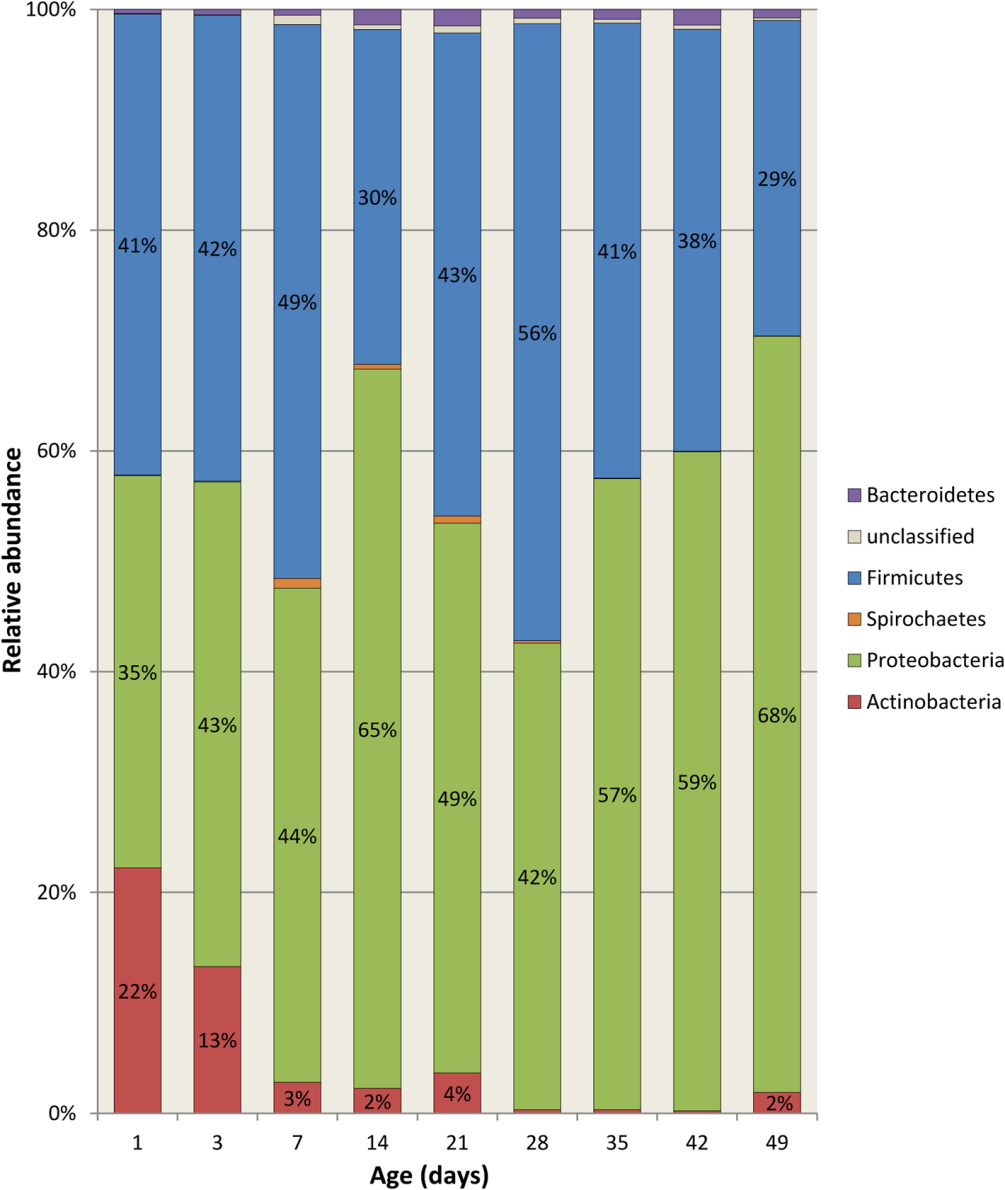
Age-associated change in relative abundance of bacterial phyla from the nasal cavity of young pigs (n = 10)

Processing of 90 nasal samples yielded a total of 5,429,616 sequences that passed the quality control measures. There was a median of 61,323 (Range: 5,381 − 104,886) sequences per sample. During the 7-week period, the nasal microbiota of 10 pigs yielded sequences that clustered into 49,458 OTUs. There was a median of 6,257 OTUs recovered per pig throughout the period of study (Range: 5,133–7,233). The OTUs were classified into 22 bacterial phyla and 676 genera. However, only three phyla had >1 % overall relative abundance: Proteobacteria (51.5 %), Firmicutes (41.0 %), and Actinobacteria (5.1 %). Only 0.38 % of sequences were unclassified at the phylum level. The temporal shift in the relative abundance of bacterial phyla in the nasal microbiota is presented in Fig. 6. Aging was associated with a reduction in Actinobacteria (*P* < 0.001) and an increase in Proteobacteria (*P* < 0.001), although a disruption in this trend was observed around the time of weaning causing the relative abundance of Proteobacteria to rebound. The dominant genus at each phase of development was *Moraxella*, with an average relative abundance ranging between 24.2 and 56.5 %. Additionally, a high relative abundance of *Clostridium* (10.4 − 16.5 %) was present before weaning but a shift in the microbiota after weaning led to an increased abundance of *Lactobacillus* (11.1 − 21.2 %) and a corresponding decrease in *Clostridium* (Table 1).

A random subsample of 5,381 sequences was used for subsequent analysis. Median sample richness was 1,152 OTUs (Range: 467–5,793), and no age-related association was observed (ρ = 0.15, *P* = 0.164). The median inverse Simpson’s diversity index was 16.7 (Range: 6.7–72.7) and, unlike richness, there was a positive correlation with age (ρ = 0.22, *P* = 0.035), although aging only accounted for a small proportion of the variation in diversity. A summary of the number of observed and core OTUs is shown in Fig. 2, and the age-related change in richness and diversity is presented in Fig. 3.

**Fig. 7:**
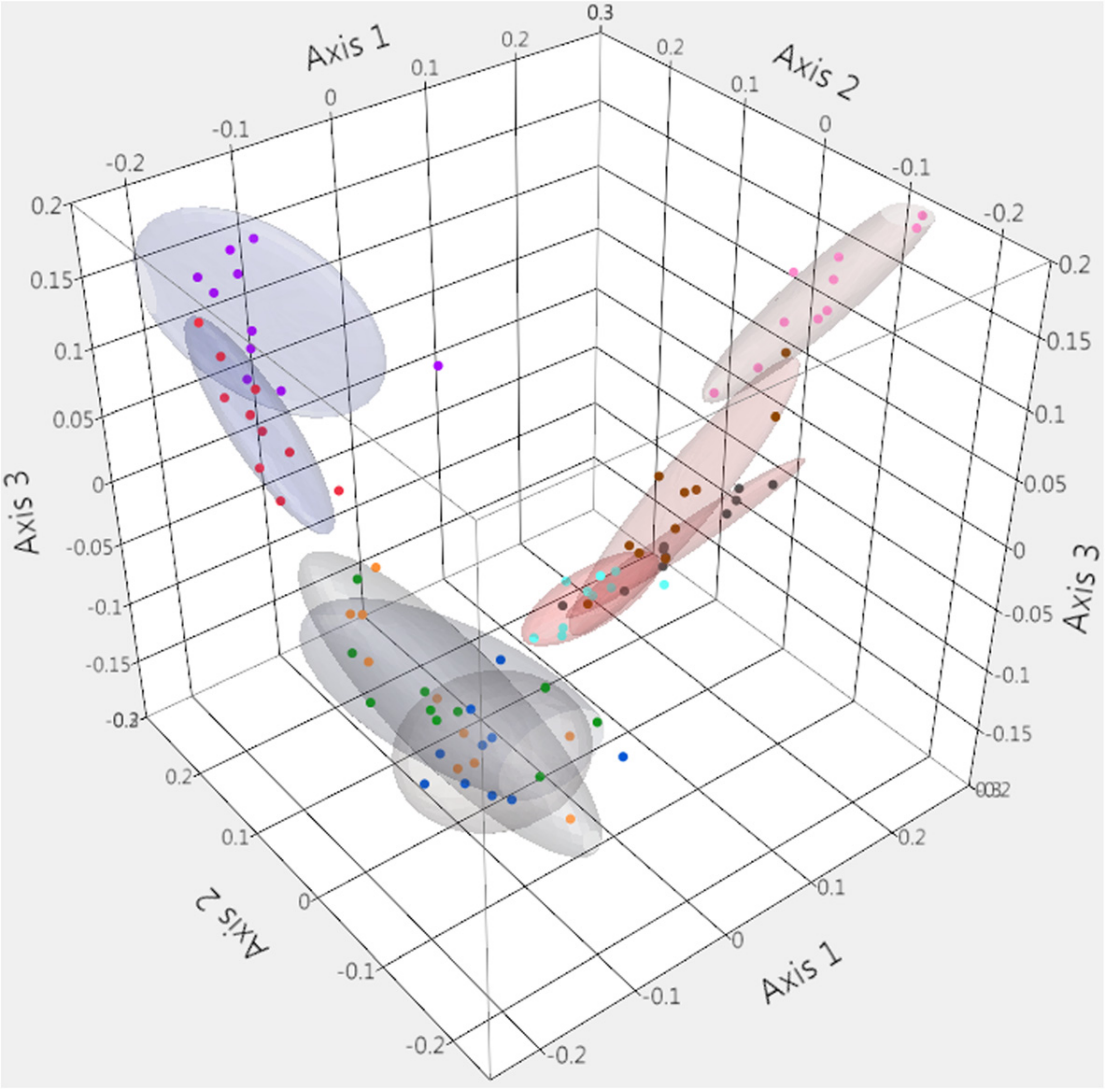
Three-dimensional principal coordinates analysis of the community membership of the porcine nasal microbiota (Jaccard Index). Coloured points and ellipses indicate age groups: 1 (red), 3 (violet), 7 (green), 14 (orange), 21 (blue), 28 (pink), 35 (brown), 42 (grey), 49 (indigo) days of age

Community membership (Jaccard measure of dissimilarity) is presented in Fig. 7. Interestingly, samples appear to cluster into 3 age-dependent categories; day 1–3, day 7–21, and day 28–49. However, when analyzed by AMOVA, the Jaccard indices differed significantly between all age groups (*P* < 0.05). Visualization of community structure (Yue and Clayton measure of dissimilarity) is displayed in Additional file 2: Figure S2 as a dendrogram. Yue and Clayton indices differed between all age groups (*P* < 0.05) with the exception of 7-day-old and 14-day-old pigs (*P* > 0.05), and 35-day-old, 42-day-old, and 49-day-old pigs (*P* > 0.05). There were also significant differences between the pre-weaning and post-weaning phases for both the Jaccard (*P* < 0.001) and Yue and Clayton indices (*P* < 0.001).

**Fig. 8:**
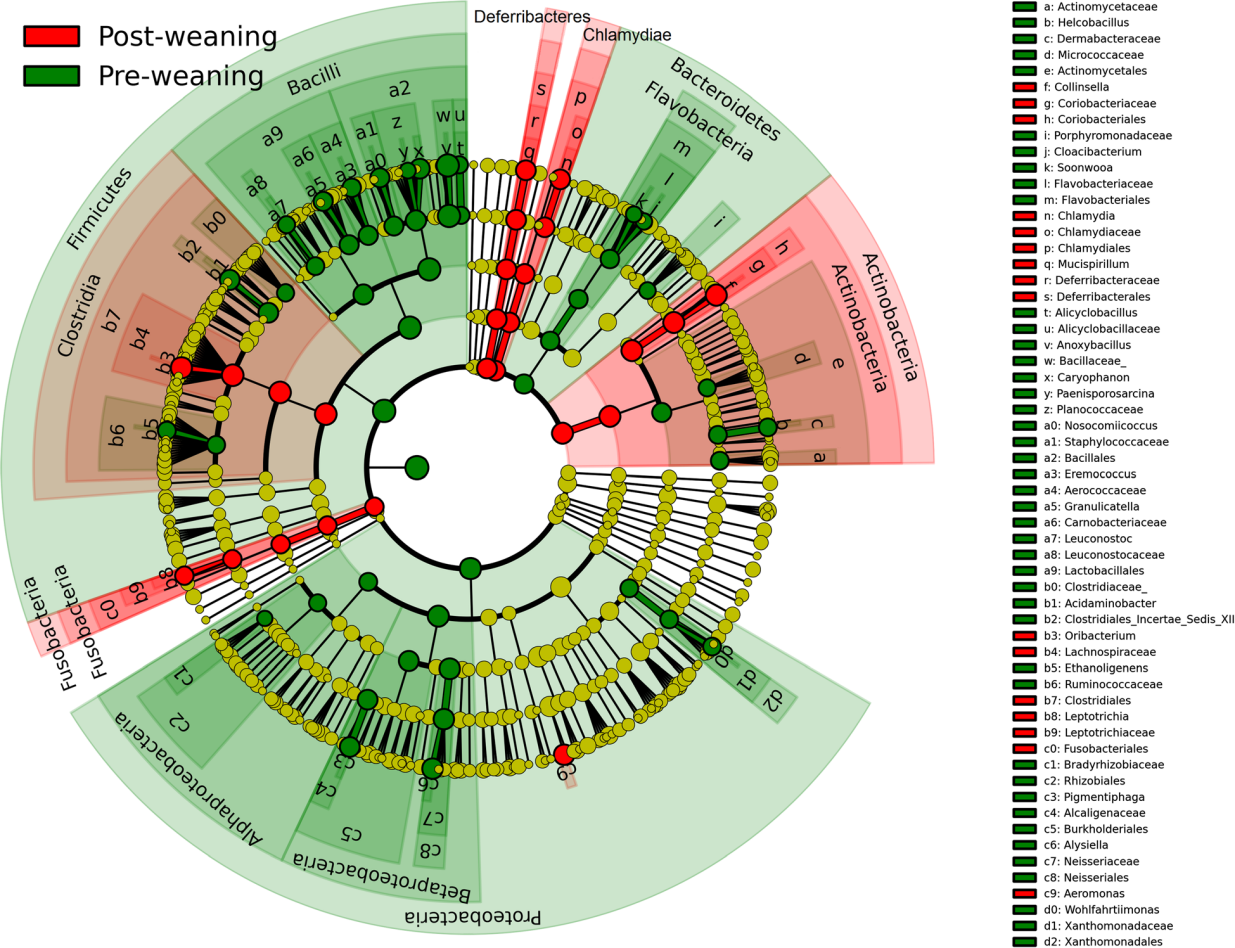
Cladogram of bacterial nasal biomarkers associated with phase of production (LEfSe). Samples were grouped into pre-weaning (days 1–21) or post-weaning (days 28–49). This hierarchal tree of taxonomical nodes, where diameter of the nodes indicates relative abundance, shows nasal biomarkers for the pre-weaning and post-weaning phases

There was a noticeable difference in the pre-weaning and post-weaning nasal microbiota. The LEfSe algorithm (LDA log score threshold = 2) demonstrated the presence of 51 OTUs characteristic to pre-weaning pigs and 25 OTUs characteristic to post-weaning pigs. The pre-weaning indicator OTUs clustered into the bacterial classes Bacilli, Flavobacteria, Alphaproteobacteria, and Betaproteobacteria, and the post-weaning indicator OTUs clustered into the classes Clostridia, Fusobacteria, Actinobacteria, Chlamydiae, and Deferribacteres (Fig. 8).

The evolving core nasal microbiota is described in Table 2. During the first 3 days of life, the genera *Haemophilus*, *Globicatella*, and *Rothia* formed the core microbiota in addition to *Clostridium sensu stricto* which was also present throughout the entire pre-weaning period. Following weaning, *Lactobacillus* was the only genus present throughout the entire nursery phase at >1 % abundance in at least 80 % of pigs.

## Discussion

The fecal and nasal microbiotas of young pigs display rich and diverse bacterial communities which undergo a very rapid and profound evolution during the first 7 weeks of life. During this developmental period a herd of pigs is exposed to thousands of bacterial species which play an important pioneering role in establishing a stable microbiota that is evident at 2–3 weeks post-weaning, particularly for the fecal microbiota. However, the instability of the microbiota during early life means it may be more susceptible to dysbiosis which can create predispositions to other illnesses as has been reported in human studies [9]. This has created the framework for the role of microorganisms in the ‘programming hypothesis’ which claims that manifestation of diseases later in life can be predetermined by exposure to environmental stimuli during early life [10]. Some research with swine have demonstrated that antimicrobials, excessive hygiene, stress, and management practices have a long-term impact on the gut microbiota [5–7, 11, 12] which may contribute to disease susceptibility or resistance. For example, it has been suggested that the abundance of lactobacilli may influence host immunity and disease susceptibility due to the immunomodulatory activity of these bacteria [13]. Further research exploring the relationship between the microbiota and the early-life programming of health in swine is warranted.

The fecal microbiota described in the present study is similar to previous studies which have found Firmicutes and Proteobacteria to be the predominant phyla pre-weaning [11, 12]. However, studies of the fecal microbiota at 3-months of age demonstrate that a taxonomical shift results in the predominance of Firmicutes and Bacteroidetes [8, 14]. The beginning of this shift was also observed in the present study; Proteobacteria are very dominant during early life (39 % relative abundance) but undergo a considerable decline into adulthood (5 % relative abundance), and the transition into adulthood is marked by an establishment of core OTUs belonging to Bacteroidetes (particularly *Prevotella* spp.). Proteobacteria, mainly comprising of *Escherichia*, are common early colonizers of the colon and the early decline in this taxa is possibly due to increased competition from obligate anaerobes (*Clostridium*, *Megasphaera*) and colonization resistance due to the immunological properties of colostrum [15].

Some notable OTUs that were abundant and formed the core microbiota after weaning belonged to butyrate-producing genera including *Megasphaera*, *Butyricicoccus*, and *Roseburia*. Butyrate is an essential energy source for enterocytes and it supports gut health by inhibiting inflammation, reducing oxidative stress, and promoting gut-barrier defense [16]. Additionally, the predominance of *Megasphaera* after weaning may be related to its extensive metabolic capabilities which include the production of vitamins, amino acids, and short-chain fatty acids [17].

The microbiota of the anterior nasal passage of young pigs described in the current investigation demonstrates similarities to the nasal microbiota of adult pigs that has previously been studied using next-generation sequencing methods [7]. During early-life, there was an apparent co-dominance between Proteobacteria and Firmicutes, but after weaning there was a noticeable shift in dominance towards Proteobacteria which is also the predominant phylum observed in adult pigs [7]. This transition appeared to coincide with weaning and was marked by an increase in Gammaproteobacteria (particularly *Moraxella*) and a decrease in Clostridium and Bacilli. The genus *Moraxella* is of particular interest due to its high abundance in pigs throughout life. Previous research has found that *Moraxella* is inversely associated with *Haemophilus* colonization in humans [4]. It is possible that *Moraxella* plays a protective role in the nasal cavity of pigs by preventing colonization with certain swine pathogens (such as *Haemophilus parasuis*); although this requires much further research.

Aging appeared to be the most significant driver of development of the fecal and nasal microbiotas, although weaning also played an important role. The reported changes in richness, diversity, relative abundance, and community structure and membership all appeared to follow trends that were independent of weaning, but that is not to say weaning was insignificant. The microbiota did respond to this event with noticeable changes in core OTUs and the detection of specific biomarker OTUs. However, this study was unable to determine whether these changes at weaning were associated with the change in diet, mixing of pigs, stress, or other variables encountered during this event. Furthermore, the rapidly evolving fecal microbiota of young pigs appears to reach a developmental milestone at approximately 2–3 weeks post-weaning. This is demonstrated by the decelerating change in relative abundance of taxa, the clustering of community membership and structure, the plateauing of richness and diversity, and the change in core OTUs. This is less evident for the nasal microbiota, but there are still indications of stability or convergence towards stability during this period.

The present characterization of the early-life microbiota of conventionally-raised pigs using next-generation sequencing provides a broader view of the microbial landscape than previous studies that have employed culture-based methods, fingerprinting, Sanger sequencing, microarrays, and 454-sequencing [1, 2, 5, 11, 12, 14] and is novel in using next-generation sequencing technology to characterize the early-life nasal microbiota, which is poorly described in the literature. However, one of the limitations of the present study is over- or under-representation of particular taxa due to amplification bias. This study also involved only healthy pigs with no clinical presentation of disease and the study was conducted at only one farm, so inter-farm variability and the impact of farm environment and management could not be assessed. Additionally, the fecal microbiota is only representative of the distal gastrointestinal tract (colon, rectum) and characterization of important bacteria populations from the stomach and small intestine may be absent or under-represented.

## Conclusions

The fecal and nasal microbiotas of pigs undergo a remarkable evolution during the first 7 weeks of life. The gastrointestinal and respiratory tracts are exposed to thousands of bacteria species with significant turn-over in community membership and structure until a relative degree of stability is evident at 2–3 weeks post-weaning. Future research should explore the significance of variation in the microbial population ecology during early-life and its implication for pathogen shedding and disease susceptibility in both young and adult pigs.

## Methods

### Study design

The use of animals in this study was approved by the Animal Care Committee at the University of Guelph. Ten conventionally-raised Yorkshire-Landrace pigs (4 males, 6 females) at a 300-sow batch-farrowing facility were enrolled on the day of birth from ten different litters (1 pig per litter). Each pig in the herd had a unique identifier assigned at birth and the pigs enrolled in the present study were randomly selected (random number generator) from a stratified sampling frame of pigs that weighed ≥1 kg at birth and did not demonstrate clinical signs of disease. Each pig was raised with their mother and litter-mates for 21 days without creep feed and, at weaning, randomized into 4 pens (located in the same nursery room) containing 11–13 pen-mates. Pigs were given a first phase ration for 14 days followed by a second phase ration for the remainder of the nursery period (Additional file 3: Table S1). The rations did not contain antimicrobial compounds and pigs were not administered any antimicrobials during the trial.

Samples were collected on days 1, 3, 7, 14, 21 (prior to weaning), 28, 35, 42, and 49. Samples were collected at the same time of day by the same investigator. At each sampling, a swab of the rectum was collected, as well as whole feces when possible, and a swab of the interior of both nares (approximately 10 mm penetration). Samples were transported at 4 °C and stored at −80 °C before being processed.

### DNA extraction

Extraction of DNA from fecal and nasal samples was completed using a commercially prepared kit and following the manufacturer’s protocol for stool DNA extraction for pathogen detection (E.Z.N.A. Stool DNA Kit, Omega Bio-Tek Inc., Doraville, Georgia, USA). For nasal samples, the whole tip of the swab was processed through the lysis stage of extraction.

### Amplification and sequencing of bacterial 16S rRNA gene

The V4 region of the 16S rRNA gene was amplified using forward (5′-AYTGGGYDTAAAGNG-3′) and reverse (5′-TACNVGGGTATCTAATCC-3′) primers previously designed [18]. The 16S primers contained adapter regions (Forward: TCGTCGGCAGCGTCAGATGTGTATAA-GAGACAG, Reverse: GTCTCGTGGGCTCGGAGATGTGTATAAGAGACAG) for annealing to Illumina universal index sequencing adaptors that were added in a later PCR.

Amplification of the 16S rRNA V4 region was completed in a 25 μl reaction consisting of 12.5 μl of KAPA 2G Fast HotStart ReadyMix 2X (KapaBiosystems), 9.0 μl of molecular-grade water, 2.5 μl template DNA, and 0.5 μl each of both the forward and reverse 16S rRNA V4 primers (10.0 μM). The reaction conditions for PCR were 94 °C for 10 min, and 27 cycles of 94 °C for 45 s, 53 °C for 60 s, and 72 °C for 90 s, followed by a final period of 72 °C for 10 min. The product was then purified using AMPure X (Beckman Coulter Inc, Mississauga, Ontario, Canada). The AMPure (20 μl) was mixed with the amplicon (25 μl) and incubated at room temperature for 2 min. After applying a magnetic field, the supernatant was discarded and the beads were washed twice with 80 % ethanol. The beads were then incubated at room temperature for 10 min before eluting with 50 μl of 10 mM Tris buffer.

Illumina universal adapters (Forward: AATGATACGGCGACCACCGAGATCTACAC-index-TCGTCGGCAGCGTC, Reverse: CAAGCAGAAGACGGCATACGAGAT-index-GTCTCGTGGGCTCGG) were then added to the purified 16S rRNA gene product by PCR using a 25 μl reaction consisting of 12.5 μl KAPA 2G Fast HotStart ReadyMix 2X (KapaBiosystems), 8.0 μl of molecular-grade water, 2.5 μl template DNA, and 1.0 μl each of the forward and reverse sample-specific Illumina universal adapters. The PCR conditions were as follows; a single cycle of 94 °C for 3 min, 8 cycles of 94 °C for 45 s, 50 °C for 60 s, and 72 °C for 90 s, followed by a final cycle of 72 °C for 10 min. The product was purified with AMPure X (Beckman Coulter Inc, Mississauga, Ontario, Canada) as previously described and DNA was eluted into 30 μl of 10 mM Tris buffer. The samples were then quantified by spectrophotometry (Nanodrop, Roche, Mississauga, Canada) and normalized to a final concentration of 2 nM. Sequencing of the library pool was performed using an Illumina MiSeq (San Diego, USA) and 2 × 250 chemistry at the University of Guelph’s Advanced Analysis Centre.

### Analysis of sequencing data

Sequencing data were analyzed using the mothur software package v.1.33.0 [19]. The paired-end reads were aligned and screened to remove sequences with the following irregularities: contiguous sequence lengths >245 bp or <239 bp, ambiguous base calls, stretches of homopolymers >8 bp, and misalignment with the target region. Sequences were then screened for chimeras using the UCHIME tool [20] and sequences belonging to non-bacterial domains, including chloroplasts, mitochondria, Archaea and Eukaryotes, were removed. Operational taxonomic units (OTUs) were created using a 3.0 % dissimilarity threshold and average neighbour algorithm, then assigned taxonomy using the RDP reference database (http://rdp.cme.msu.edu). The observed relative abundance of taxa was analyzed prior to subsampling and the mean relative abundance of the 10 pigs was plotted graphically and in a chart to demonstrate changes in phyla, classes, and genera over time. Random subsampling of sequences from each individual sample was completed to normalize the sequence count. Community diversity (inverse Simpson index) and richness (CatchAll; [21]) were computed and sampling coverage was assessed by Good’s coverage value. The core microbiota (OTUs with >1 % relative abundance and shared amongst ≥80 % of pigs) was also explored for each sample type at different ages. The Jaccard index of dissimilarity was used as an assessment of community membership and the Yue and Clayton measure of dissimilarity was used to as an assessment of community structure. These indices were visualized by principle coordinate analysis, and compared between groups using analysis of molecular variance (AMOVA). Probabilistic modelling using Dirichlet multinomial mixtures (DMM) was utilized in examining community types associated with phase of production [22]. Additionally, the linear discriminant analysis (LDA) effect size (LEfSe) algorithm was used to identify OTUs associated with pre-weaning and post-weaning microbiotas [23] and cladograms were produced using the online LEfSe tool (http://huttenhower.sph.harvard.edu/galaxy/). Non-parametric statistical analysis (Spearman’s rank correlation) was performed in STATA 10.0 I/C (Stata Corporation, College Station, TX) to determine age-related differences in taxonomical abundance, diversity, and richness. The null hypothesis for all statistical tests was rejected at *P* < 0.05.

## Availability of data and materials

Raw sequences were uploaded to the Sequence Read Archive under study number SRP059335 (experiment number SRX1055388).

## Additional files

Additional file 1: Figure S1. Dendrogram of the community structure of the porcine fecal microbiota (Yue and Clayton). Sample identification (ABBCC) coded as: A – fecal (F) or nasal (N) sample, BB – age of pig, CC – unique pig identifier. (PNG 31 kb) Additional file 2: Figure S2. Dendrogram of the community structure of the porcine nasal microbiota (Yue and Clayton). Sample identification (ABBCC) coded as: A – fecal (F) or nasal (N) sample; BB – age of pig; CC – unique pig identifier. (PNG 33 kb) Additional file 3: Table S1. Two-phase starter ration fed to nursery pigs. (PDF 56 kb)
