# Supplementary figures and images for: Longitudinal study of the early-life fecal and nasal microbiotas of the domestic pig
Source: BMC Microbiol. 2015 Sep 21;15:184. doi: 10.1186/s12866-015-0512-7 (PMC4578254; doi:10.1186/s12866-015-0512-7)

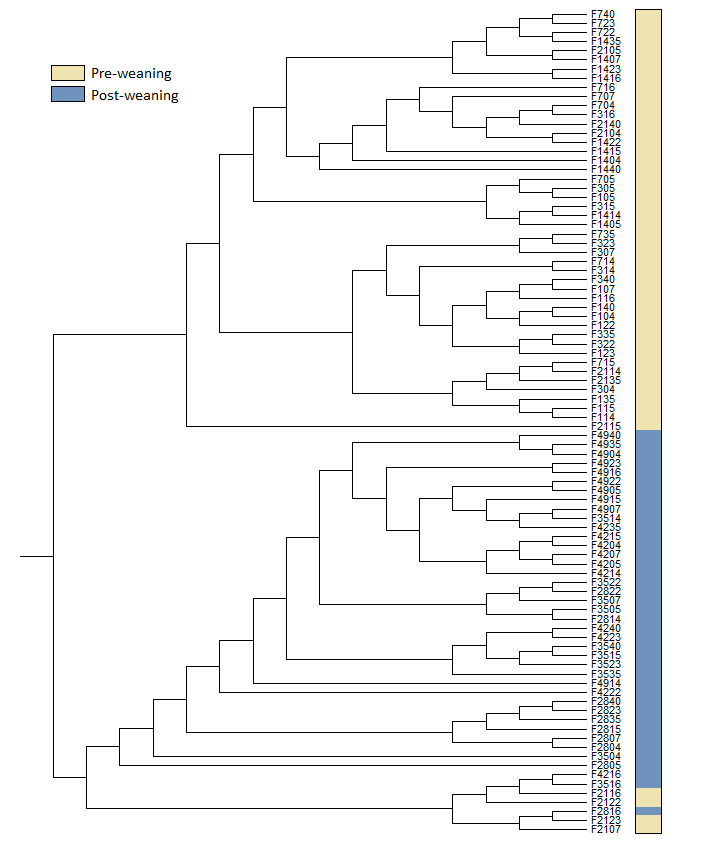

Supplement: Additional file 1: Figure S1. — Dendrogram of the community structure of the porcine fecal microbiota (Yue and Clayton). Sample identification (ABBCC) coded as: A – fecal (F) or nasal (N) sample, BB – age of pig, CC – unique pig identifier. (PNG 31 kb) [file 12866_2015_512_MOESM1_ESM.png]

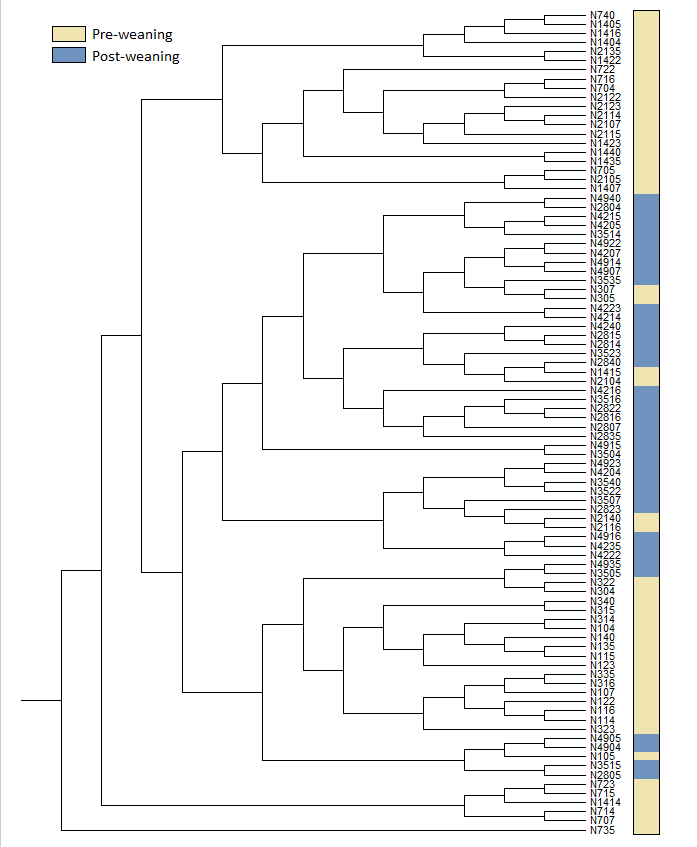

Supplement: Additional file 2: Figure S2. — Dendrogram of the community structure of the porcine nasal microbiota (Yue and Clayton). Sample identification (ABBCC) coded as: A – fecal (F) or nasal (N) sample; BB – age of pig; CC – unique pig identifier. (PNG 33 kb) [file 12866_2015_512_MOESM2_ESM.png]
