# Supplementary material for: Longitudinal study of the early-life fecal and nasal microbiotas of the domestic pig
Source: BMC Microbiol. 2015 Sep 21;15:184. doi: 10.1186/s12866-015-0512-7 (PMC4578254; doi:10.1186/s12866-015-0512-7)
Supplement: Additional file 3: Table S1. — Two-phase starter ration fed to nursery pigs. (PDF 56 kb) [file 12866_2015_512_MOESM3_ESM.pdf]

Supplementary Table 1. Two-phase starter ration fed to nursery pigs.

**Ingredients**	**Composition (%)**	
	**Phase I Ration**	**Phase II Ration**
Soybean meal, 47%	25.5	25.8
Corn chop	35.3	33.5
Whey permeate	10	8
Bakery meal	10	10
Wheat chop	7.8	7.5
Wheat shorts	0	5
Fish meal	3	2
Blood meal	2.5	2.5
Tallow	1.4	1.5
Monocalcium phosphate	1.1	1.05
Limestone	0.95	1
L-lysine, 50%	0.62	0.55
DL-methionine, 88%	0.28	0.25
Maple Butter	0.2	0.1
Fine salt	0.2	0.13
Threonine	0.19	0.16
Selenium	0.1	0.1
Choline chloride, 70%	0.05	0.05
Vitamin E (50 KIU/kg)	0.05	0.05
PellTech	0.4	0.4
Integral	0.05	0.05
Superzyme-CS	0.05	0.05
IntelliBond C (58% Cu)	0.017	0.017
Vitamin and mineral supplement[a]	0.2	0.2

[a] Formulation per kilogram of starter ration: 100 mg zinc oxide, 270 mg iron, 28 mg manganese, 124 mg copper, 0.60 mg iodine, 0.50 mg selenium, 0.16 mg cobalt, 20 mg fluorine, 10 KIU vitamin A, 1.5 KIU vitamin D3, 70 IU vitamin E, 2.0 mg vitamin K, 20 µg vitamin B12, 6.5 mg riboflavin, 30 mg niacin, 1.34 g choline, 24 mg D-pantothenic acid, 2.5 mg pyridoxine, 2.0 mg thiamine, 3.0 mg folic acid, 200 µg biotin.
